# Long read reference genome-free reconstruction of a full-length transcriptome from *Astragalus membranaceus* reveals transcript variants involved in bioactive compound biosynthesis

**DOI:** 10.1038/celldisc.2017.31

**Published:** 2017-08-29

**Authors:** Jun Li, Yuka Harata-Lee, Matthew D Denton, Qianjin Feng, Judith R Rathjen, Zhipeng Qu, David L Adelson

**Affiliations:** 1 Department of Genetics and Evolution, School of Biological Sciences, The University of Adelaide, Adelaide, SA, Australia; 2 Zhendong Center for the Molecular Basis of Traditional Chinese Medicine, School of Biological Sciences, The University of Adelaide, Adelaide, SA, Australia; 3 School of Agriculture, Food and Wine, The University of Adelaide, Adelaide, SA, Australia; 4 Shanxi Modern Chinese Medicine Engineering Laboratory, Shanxi University of Traditional Chinese Medicine, Shanxi, China

**Keywords:** *Astragalus membranaceus*, transcriptome, Iso-Seq, biosynthesis, alternative splicing

## Abstract

*Astragalus membranaceus,* also known as Huangqi in China, is one of the most widely used medicinal herbs in Traditional Chinese Medicine. Traditional Chinese Medicine formulations from *Astragalus membranaceus* have been used to treat a wide range of illnesses, such as cardiovascular disease, type 2 diabetes, nephritis and cancers. Pharmacological studies have shown that immunomodulating, anti-hyperglycemic, anti-inflammatory, antioxidant and antiviral activities exist in the extract of *Astragalus membranaceus*. Therefore, characterising the biosynthesis of bioactive compounds in *Astragalus membranaceus*, such as Astragalosides, Calycosin and Calycosin-7-O-β-d-glucoside, is of particular importance for further genetic studies of *Astragalus membranaceus*. In this study, we reconstructed the *Astragalus membranaceus* full-length transcriptomes from leaf and root tissues using PacBio Iso-Seq long reads. We identified 27 975 and 22 343 full-length unique transcript models in each tissue respectively. Compared with previous studies that used short read sequencing, our reconstructed transcripts are longer, and are more likely to be full-length and include numerous transcript variants. Moreover, we also re-characterised and identified potential transcript variants of genes involved in Astragalosides, Calycosin and Calycosin-7-O-β-d-glucoside biosynthesis. In conclusion, our study provides a practical pipeline to characterise the full-length transcriptome for species without a reference genome and a useful genomic resource for exploring the biosynthesis of active compounds in *Astragalus membranaceus*.

## Introduction

*Astragalus membranaceus*, a member of the legume family, is one of the most commonly used herbs in Traditional Chinese Medicine (TCM) [1]. *A. membranaceus*, also known as ‘Huangqi’ (specifically refers to the dried root of *A. membranaceus*), is mainly grown in the northern regions of China as well as in Mongolia and Korea [2]. *A. membranaceus* has been shown to possess immunomodulating, anti-hyperglycemic, anti-inflammatory, antioxidant and antiviral activities [3]. There has also been a long history of the clinical use of products from *A. membranaceus* as adjuvant therapies in the treatment of cardiovascular illness, type 2 diabetes, nephritis and cancers in China [2,3,4]. The major bioactive components of *A. membranaceus* root extract are triterpenoid saponins, flavonoids and polysaccharides [2, 4,5,6,7]. Specifically, the pharmacological effects of Astragalosides (AST), Calycosin and Calycosin-7-O-β-d-glucoside (CG) have been well-studied [8,9,10,11,12]. To provide resources for future molecular barcoding and plant breeding, we have to understand the biosynthetic pathways of these bioactive compounds and characterise the corresponding key genes important for *A. membranaceus*-related TCM formulations.

The first step for investigating the biosynthetic pathways of bioactive compounds in *A. membranaceus* is to obtain the nucleotide sequences of genes. Whole-genome sequencing and assembly, combined with transcriptome data would be the ideal way to systematically characterise gene models. However, high sequencing costs because of the necessity for both long read and high-depth short read sequences for accurate *de novo* assembly put this beyond the reach of smaller research labs. This is especially true for many plants with large genomes, for example the well-annotated crop legume, Soybean (*Glycine Max*), which has a genome size of ~1.1 Gb [13]. Genome assembly is also complicated by polyploidy, such as for wheat (*Triticum aestivum*) [14], and by a high proportion of repetitive elements, such as for maize (*Zea mays*) [15]. Therefore, characterising gene models based on transcriptome data is a faster and more economical approach for species without a reference genome. Homology-based cDNA cloning was initially used to amplify full-length sequences of *A. membranaceus* genes from different biosynthetic pathways. *AmPAL1* (encoding Phenylalanine ammonia lyase), *AmCHS* (encoding Chalcone synthase), *AmIFS* (encoding Isoflavone synthase), *AmI3’H* (encoding Isoflavone 3′-hydroxylase) and *AmUCGT* (encoding Calycosin-7-O-glucosyltransferase) were cloned and characterised with respect to their roles in the biosynthetic pathways of Calycosin and CG [16,17,18]. Recently, gene characterisation at transcriptome scale was carried out in *A. membranaceus* with high-throughput next-generation sequencing in which 9 893 UniGenes were identified and a number of genes related to secondary metabolite production were characterised, by using the 454 sequencing platform [19]. In addition, another transcriptome study using the Illumina sequencing platform was carried out on the hairy roots of *A. membranaceus* and identified genes involved in AST, Calycosin and CG biosynthesis [20]. However, although next-generation sequencing can achieve a very high throughput for transcriptomes, the limitation of short read length restricts the yield of full-length genes, which can be overcome by using third-generation long read sequencing.

Third-generation sequencing platforms, such as single-molecule real-time (SMRT) sequencing from PacBio can generate long reads of up to 60 kb, with half of them longer than 20 kb (with P6-C4 chemistry). Isoform sequencing (Iso-Seq), which is based on the SMRT sequencing platform, has been used to analyse full-length transcriptomes in multiple plant species [21,22,23,24]. For example, whole-genome wide gene annotation was validated and improved using SMRT reads for sugar beet (*Beta vulgaris*), spinach (*Spinacia oleracea*) and wheat (*T. aestivum*) genomes [21,22]. The complexity of maize (*Z. mays*) and sorghum (*Sorghum bicolour*) transcriptomes, particularly for abundant alternative splicing (AS) events, was also investigated using PacBio Iso-Seq [23,24]. Finally, Iso-Seq has also been applied to analyse the full-length transcriptome of Danshen (*Salvia miltiorrhiza* Bunge), which is also a widely used medicinal plant in TCM [25].

To analyse the full-length transcriptome of *A. membranaceus*, we used PacBio Iso-Seq to generate comprehensive full-length transcriptomes from leaf and root tissues. We then systematically carried out functional annotation of those full-length transcriptomes. Isoform analysis revealed the complexity of AS in *A. membranaceus* and allowed us to detect different transcription isoforms from different tissues. Moreover, long-noncoding RNAs (lncRNAs) were identified and genes involved in biosynthesis of major active compounds, for example AST, Calycosin and CG, were also re-characterised. Herein, we not only systematically characterised the complexity of the *A. membranaceus* full-length transcriptome, but we also provide a valuable resource for investigating the biosynthesis of important bioactive compounds in *A. membranaceus*.

## Results

### *A. membranaceus* transcriptome analysis using PacBio Iso-Seq

The full-length transcriptome of *A. membranaceus* was generated using PacBio Iso-Seq on leaf and root tissues. For leaf tissue, we obtained 494 408 reads of inserts (ROI) from a total of eight SMRT cells, including 218 611 ROIs from three SMRT cells for 1–2 kb fragments and 275 797 ROIs from five SMRT cells for 2–3 kb fragments. Another 500 007 ROIs were acquired from eight SMRT cells for root tissue, including 219 823 ROIs from three SMRT cells for 1–2 kb fragments and 280 184 ROIs from five SMRT cells for 2–3 kb fragments (Supplementary Table S1). The average lengths of ROIs were 2 409 and 2 461 nucleotides (nt) for leaf and root tissues, respectively (Supplementary Table S1).

**Figure 1:**
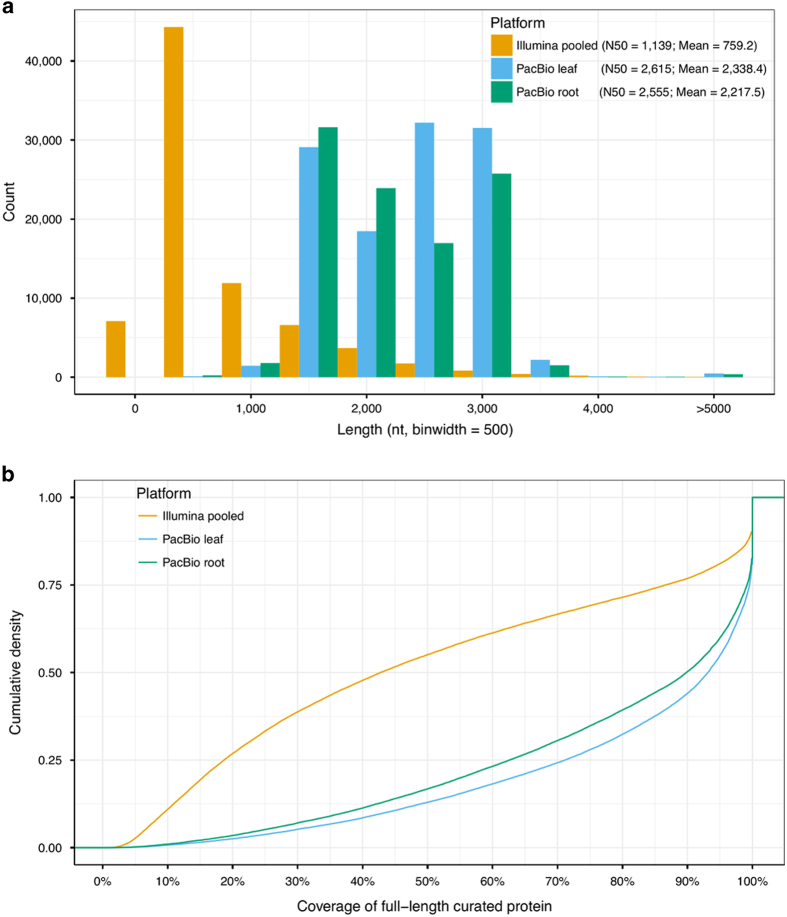
Comparison of *A. membranaceus* transcriptomes acquired using different sequencing platforms. (**a**) Length distribution of Iso-Seq consensus transcripts compared with *de novo* assembled transcripts from Illumina sequencing platform. N50 length similar to the median contig or assembled sequence length and is defined as the length *N* for which 50% of all bases in the assembled sequences (either Illumina *de novo* assembled transcript or Iso-Seq consensus transcript) are in an assembled sequence of length *L*<*N*. (**b**) Cumulative density plot showing the coverage of full-length curated proteins (UniProtKB) for transcripts identified by different sequencing platforms.

By applying the standard Iso-Seq classification and clustering protocol (see Materials and Methods) on the above ROIs, we produced 115 725 full-length consensus transcripts, including 75 816 polished high-quality (HQ) and 39 909 low-quality (LQ) transcripts for leaf tissue. For root tissue, we generated 102 334 full-length consensus transcripts, including 73 755 polished HQ and 28 579 LQ transcripts (Table 1). The N50 (N50 length similar to the median contig or assembled sequence length and is defined as the length *N* for which 50% of all bases in the assembled sequences are in an assembled sequence of length *L*<*N* [26].) of full-length consensus transcripts from Iso-Seq was double those of assembled transcripts from 454 and Illumina next-generation sequencing (Figure 1a, N50=1 205 nt for 454 assembled transcripts according to reference [19].). To compare the transcripts produced by different sequencing platforms, we looked at complete open reading frames (ORFs) from Iso-Seq consensus transcripts and Illumina *de novo* assembled transcripts aligned against a well-curated full-length protein database, UniProt Knowledgebase (UniProtKB). Compared with Illumina *de novo* assembled transcripts, a significantly higher percentage of Iso-Seq consensus isoforms contained full-length ORFs (covered 100% of curated full-length protein) or near full-length ORFs (covered >80% of curated full-length protein) (Figure 1b). These results demonstrate that PacBio Iso-Seq is an efficient strategy to generate HQ full-length transcripts without assembly, an element of critical importance for genomic studies on species without a reference genome assembly.

### Reconstruction of unique full-length transcript models of *A. membranaceus* without a reference genome

**Figure 2:**
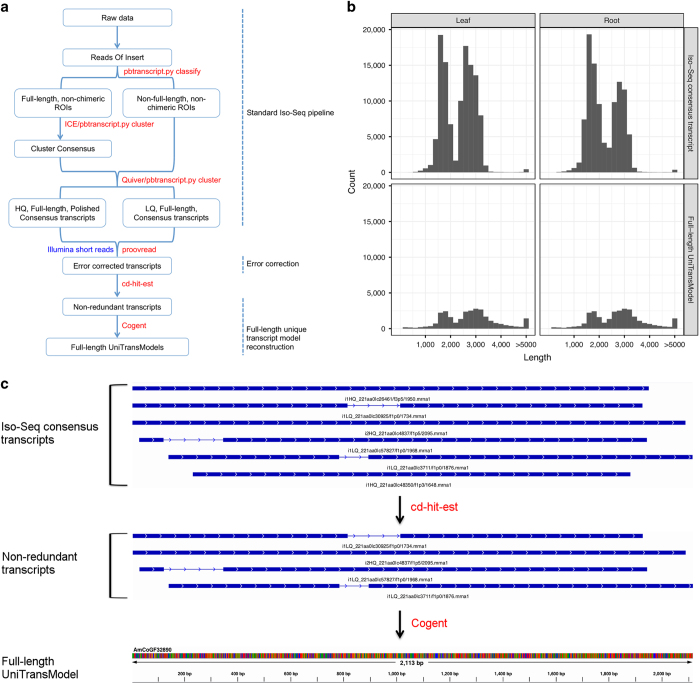
Reconstruction of *A. membranaceus* full-length UniTransModels from Iso-Seq. (**a**) Pipeline used for reconstruction of full-length UniTransModels from Iso-Seq without reference genome (see Materials and Methods for details). (**b**) Length distribution of Iso-Seq consensus transcripts (standard Iso-Seq pipeline) and full-length UniTransModels (full pipeline). (**c**) Example showing two steps of the pipeline for full-length UniTransModel reconstruction. Six Iso-Seq consensus transcripts (top panel) were reduced to four non-redundant transcripts (middle panel) owing to redundancy, and then one unique sequence (bottom panel) was reconstructed as the UniTransModel from the four non-redundant transcripts. Blocks in blue represent the exons and lines in-between represent introns in each transcript.

To generate a data set of unique full-length transcript models from Iso-Seq reads for *A. membranaceus* without a reference genome, we developed a computational pipeline that leveraged several publicly available tools (Figure 2a). After consensus transcripts were generated with the standard Iso-Seq bioinformatics pipeline, we error corrected both the HQ and LQ consensus transcripts using ‘proovread’ and publicly available Illumina short reads (Accession number: ERX651043, DDBJ) [27]. Redundant error-corrected transcripts were subsequently removed by applying ‘cd-hit-est’ with optimised parameters (see Materials and Methods). COding GENome reconstruction Tool (Cogent) was then used to further partition these error-corrected non-redundant transcripts into transcript families and reconstruct each family into one or several full-length unique transcript model(s) (referred to as UniTransModel) based on k-mer clustering and De Bruijn graph methods. By applying this pipeline to our *A. membranaceus* Iso-Seq data, we obtained 27 975 and 22 343 full-length UniTransModels for leaf and root tissues, respectively. We observed similar length distributions for full-length UniTransModels compared with consensus transcripts from the standard Iso-Seq bioinformatics pipeline, but the numbers of transcripts ~1 600 and ~2 800 nt long were significantly reduced by our pipeline (Figure 2b and c). This indicates that there was still a high level of redundancy for transcripts in the standard Iso-Seq bioinformatics pipeline output. Next, we identified the tissue-specific full-length UniTransModels in leaf or root based on their sequence similarity. About half of the UniTransModels were only detected in one of two tissues (13 105 out of 27 975 in leaf, and 11 011 out of 22 343 in root) (Supplementary Figure S1). Analysis of gene ontology (GO) over-representation of these tissue-specific UniTransModels showed that ‘small molecular metabolic process’ is the most significantly over-represented term in both tissues. In addition, genes with functions associated with different ‘metabolic process’ terms were also significantly enriched in leaf tissue, while more ‘localisation’ and ‘transport’ related function terms were significantly over-represented in root in the ‘Biological process’ category (Supplementary Figure S2). With respect to category of ‘Molecular function’, related terms such as ‘nucleotide binding’ were significantly over-represented in leaf tissue, whereas enzyme ‘activity’ related terms, for example, ‘ATPase activity’ and ‘nucleoside-triphosphatase activity’, were significantly over-represented in root tissue (Supplementary Figure S2).

### Functional annotation of full-length *A. membranaceus* transcriptomes from two tissues

To obtain a comprehensive functional annotation of the *A. membranaceus* transcriptome, we annotated full-length UniTransModels from each tissue by similarity search against protein sequences curated in UniProtKB and soybean reference proteins (Supplementary Table S2). A total of 96.7% and 96.2% of UniTransModels in leaf and root were annotated with significant hits in these well-curated databases (*E*-value⩽1e–3) (Supplementary Table S3). The small numbers of unannotated UniTransModels (933 in leaf and 843 in root) might represent novel *A. membranaceus* species-specific genes.

**Figure 3:**
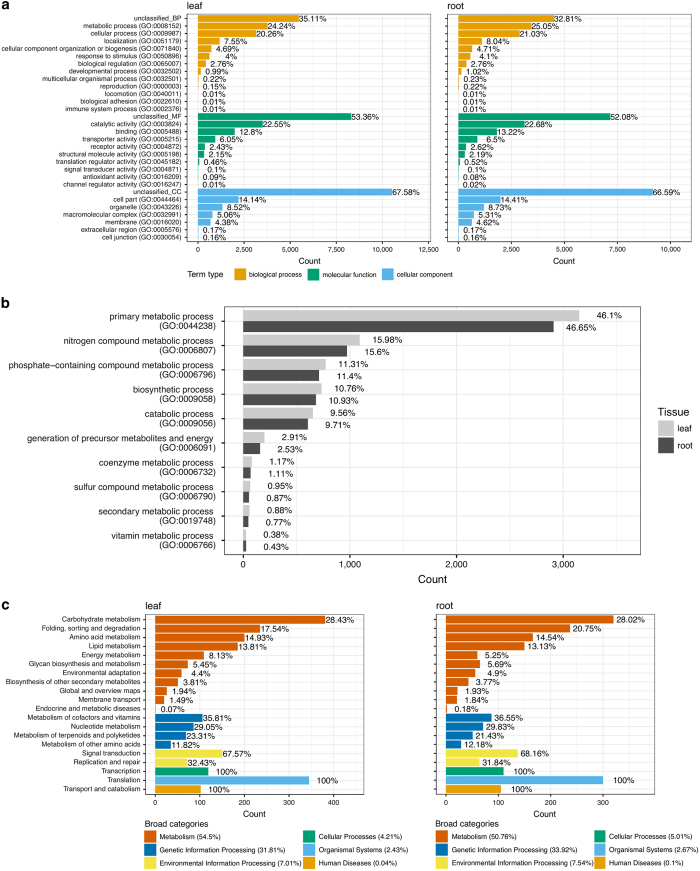
Functional annotation and classification of *A. membranaceus* UniTransModels in leaf and root. (**a**) GO classification of *A. membranaceus* UniTransModels in leaf and root. (**b**) Further classification of UniTransModels involved in ‘metabolic process’ functional terms in GO. (**c**) KEGG pathway classification of *A. membranaceus* UniTransModels in leaf and root.

As soybean has well-characterised gene annotation and functional classification based on a reference genome, we used the significant hits against soybean UniProtKB to perform functional classification for *A. membranaceus* UniTransModels. 11 046 and 9 919 soybean annotated UniTransModels were identified with different functional terms of three GO categories in leaf and root, respectively (Figure 3a). Overall, we observed very similar GO functional classifications for UniTransModels in leaf and root tissues. Among classified functional terms, ‘metabolic process’ was identified as the most common annotation for UniTransModels (~25%) in both tissues. In addition, ‘cellular process’ and ‘localisation’ were the next most abundant ‘Biological process’ GO terms in both tissues. In the ‘Molecular function’ and ‘Cellular component’ GO categories, ‘catalytic activity’ and ‘cell part’ annotations were identified as most abundant in both tissues (Figure 3a). The breakdown of UniTransModels annotated as ‘metabolic process’ indicated that almost half of them were associated with ‘primary metabolic process’ in both tissues, and ~15% of UniTransModels were involved in ‘nitrogen compound metabolic process’ (Figure 3b). Of particular interest, ~0.8% UniTransModels were classified as ‘secondary metabolic process’ in both tissues (Figure 3b).

In addition to GO functional classification, we also used the Kyoto Encyclopedia of Genes and Genomes (KEGG) to investigate the functional landscape of *A. membranaceus* UniTransModels. As with the GO classification, the percentages of different classes of KEGG pathway terms were quite similar in leaf and root. More than half of annotated UniTransModels were classified as belonging to ‘Metabolism’ related pathways, in which ‘Carbohydrate metabolism’, ‘Folding, sorting and degradation’ and ‘Amino acid metabolism’ were the top 3 pathways with most abundant transcripts in both leaf and root (Figure 3c). The difference between the two tissues with respect to KEGG annotation is the percentages of genes involved in ‘Folding, sorting and degradation’ and ‘Energy metabolism’. There was a lower percentage of transcripts associated with ‘Folding, sorting and degradation’ in leaf compared with root tissues (17.54% compared with 20.75%), however there was a higher percentage of transcripts associated with ‘Energy metabolism’ in leaf compared with root tissues (8.13% compared with 5.25%) (Figure 3c).

Together, the results from GO and KEGG functional annotation and classification of UniTransModels, allowed us to obtain a comprehensive functional characterisation for the full-length transcriptomes from leaf and root of *A. membranaceus*. The overall similar functional classification of transcripts in these two tissues indicates that the transcriptome at pathway level is generally conserved, although the expression of individual genes might be different.

### AS detection without a reference genome

**Figure 4:**
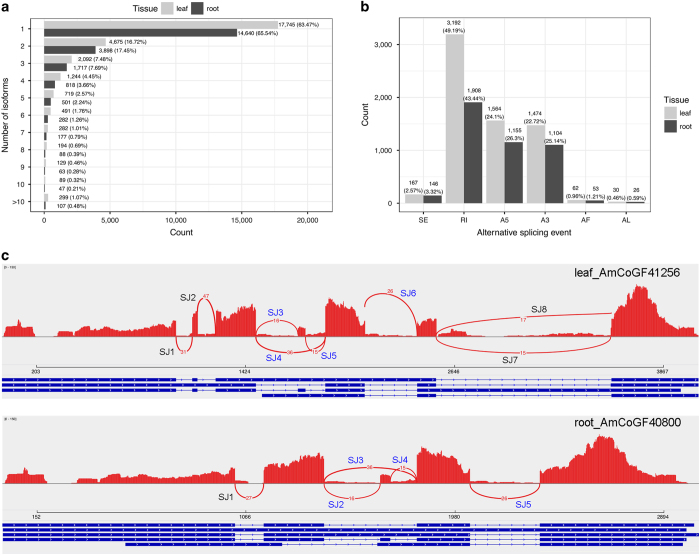
Isoform analysis of *A. membranaceus* full-length transcriptomes using Iso-Seq. (**a**) Distribution of isoform numbers for UniTransModels in leaf and root. (**b**) Numbers of different AS events detected in full-length transcriptomes from root and leaf. These AS events were detected using UniTransModels as reference, which are different to AS events based on genome sequence. SE, skipping exon; RI, retained intron; A5, alternative 5′ splice-site; A3, alternative 3′ splice-site; AF, alternative first exon; AL, alternative last exon. (**c**) Sashimi plot showing an example of the same gene generating different transcript isoforms detected with our pipeline in two tissues. There are four isoforms, including eight splicing junction sites (SJ) in leaf, and five isoforms, consisting of five SJs in root. Four SJs are shared between isoforms in two tissues (SJ3–SJ6 in leaf corresponding to SJ2–SJ5 in root, coloured in blue). Peaks in red represent the coverage of short reads and curved lines with numbers in red represent splicing junctions supported by that number of short reads. For each isoform, blocks in blue represent exons and lines in-between represent introns.

One advantage of PacBio Iso-Seq is its ability to describe the complexity of AS at whole transcriptome scale. Transcript isoforms were identified by aligning individual Iso-Seq consensus transcripts back to the reconstructed full-length UniTransModels. A total of 36.53% of full-length UniTransModels had more than one isoform in leaf and a slightly lower percentage (34.46%) in root (Figure 4a). It is interesting to note that there are many more UniTransModels with more than 10 isoforms in leaf (299, 1.07%) compared with root tissue (107, 0.48%) (Figure 4a). We described the specific types of AS events by using the UniTransModels as our reference. We denoted these AS events as UniTransModel-based instead of as canonical genome-based AS events. We identified 6 494 and 4 399 UniTransModel-based AS events in leaf and root tissue, respectively (Figure 4b). UniTransModel-based retained introns were identified as the most abundant AS event in both leaf and root. Together with alternative 5′ end or 3′ end AS events, these three types of AS event accounted for >90% of detected events (Figure 4b). By mapping Illumina short reads to transcript models, we were able to confirm the reliability of isoform detection using our pipeline, even in the absence of a reference genome (Figure 4c). We also detected different splicing isoforms of the same UniTransModels in different tissues (Figure 4c).

We then determined whether there is bias with respect to number of isoforms for genes with different functional GO annotations. At the top-level of GO classification, we observed very similar distributions for the nine GO ‘Biological process’ functional terms with >10 genes annotated in leaf, while in root, genes annotated as ‘multicellular organismal process’ have many fewer transcripts with more than one isoform compared with genes from other functional terms (Supplementary Figure S3A). For transcripts annotated with functional terms in ‘metabolic process’, we found that there were a greater number of genes annotated as ‘coenzyme metabolic process’ with more than one isoform compared with other metabolic process functional terms. Furthermore, we also found a higher percentage of genes with more than one isoform in ‘primary metabolic process’ compared with ‘secondary metabolic process’ in both leaf and root tissues (Supplementary Figure S3B).

### LncRNA identification in *A. membranaceus* full-length transcriptomes

**Figure 5:**
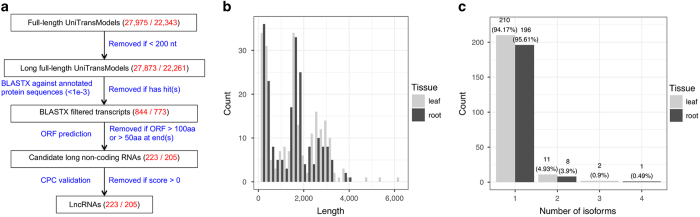
Identification of *A. membranaceus* lncRNAs. (**a**) Pipeline used to identify lncRNAs. Text in black: data sets used/processed in different steps. Text in blue: tools and thresholds used for filtering protein-coding transcripts. Text in red: numbers of transcripts in each data set, with left for leaf and right for root. (**b**) Length distribution of identified lncRNAs in leaf and root. (**c**) Distribution of isoform numbers for lncRNAs in two tissues.

We identified lncRNAs from these PacBio Iso-Seq data sets using a customised pipeline (Figure 5a) comprised of three steps: (a) removal of annotated protein-coding transcripts by BLASTX against multiple protein databases; (b) removal of potential unannotated protein-coding transcripts based on ORF length; and (c) validation of coding potential with Coding Potential Calculator [28]. We obtained 223 and 205 lncRNAs from 27 975 and 22 343 UniTransModels in leaf and root, respectively. Some of these lncRNAs were up to 4 000 nt long, particularly in leaf (Figure 5b), and most of them were single-isoform transcripts present in both tissues (Figure 5c). Only small numbers of these lncRNAs were annotated with known RNA motifs by searching against Rfam ncRNA families, and most of these appeared to be precursor transcripts of snoRNAs or miRNAs (Supplementary Table S4). The functions of these lncRNAs, particularly the unannotated ones, need to be further characterised.

### Re-characterisation of genes involved in biosynthesis of bioactive compounds in *A. membranaceus*

**Figure 6:**
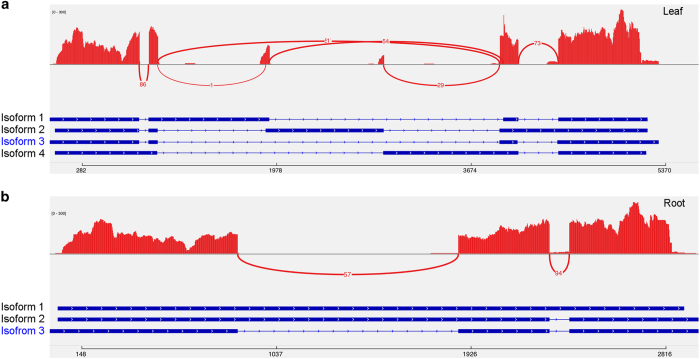
Sashimi plot showing transcript isoforms of the *AmMVD* gene (encoding mevalonate diphosphate decarboxylase) in leaf (**a**) and root (**b**). ‘Isoform 3’ in leaf and ‘Isoform 3’ in root are the same transcript isoform as the NCBI reference (Accession number: KF355964). Peaks in red represent the coverage of short reads and curved lines with numbers in red represent splicing junctions supported by that number of short reads. For each isoform, blocks in blue represent exons and lines in-between represent introns.

AST, Calycosin and CG are well-studied bioactive compounds, and key genes in their biosynthetic pathways have already been identified [20]. We searched our UniTransModels using NCBI mRNA sequences from 18 genes involved in AST biosynthesis and 14 genes involved in Calycosin and CG biosynthesis (Supplementary Tables S5 and S6). We identified 14 leaf UniTransModels and 13 root UniTransModels corresponding to 17 NCBI mRNAs in the AST biosynthetic pathway. Our Iso-Seq full-length sequences were longer compared with those 17 NCBI mRNA sequences, the majority of which (15 out of 17) had their sequences almost fully represented in Iso-Seq full-length sequences (with coverage >95%) (Supplementary Table S5). We also found that several of these genes might possess multiple transcription isoforms in both tissues. Moreover, *AmMVD* may also have tissue-specific transcription isoforms in two tissues (Figure 6 and Supplementary Figure S4). With respect to Calycosin and CG biosynthesis, we identified 11 leaf UniTransModels corresponding to 12 NCBI mRNAs and 9 root UniTransModels corresponding to 10 NCBI mRNAs, where the sequences of all of the NCBI mRNAs were almost fully represented (with coverage >95%) in either leaf or root Iso-Seq full-length UniTransModels (Supplementary Table S6). As with the AST biosynthetic pathway, generally longer sequences and multiple transcription isoforms were also found for these UniTransModels involved in Calycosin and CG biosynthesis in our full-length Iso-Seq transcriptome (Supplementary Table S6). Our full-length Iso-Seq transcriptome can provide not only additional information for re-characterisation of the biosynthesis of AST, Calycosin and CG at a deeper transcription isoform level, but will also help understand the biosynthesis of other bioactive compounds in *A. membranaceus*.

## Discussion

Transcriptome reconstruction and annotation, particularly for species without a reference genome sequence, has improved significantly in parallel with the development of sequencing techniques. Expressed sequence tags (ESTs) from cDNA libraries were the first generation of high-throughput transcriptome acquisition using Sanger sequencing technique. Most ESTs were not full-length (~500–800 bp) and although it was very time-consuming and costly to get large scale data for ESTs, transcriptome exploration based on ESTs played a critical role in gene discovery, gene structure characterisation and genome annotation in the pre-genomic and early-genomic eras [29]. RNA sequencing (RNA-seq) arose based on the development of next-generation sequencing techniques and has made transcriptome analysis useful for differential gene expression analysis, alternative gene splicing characterisation, gene mutation and single-nucleotide polymorphism identification and detection of gene fusions [30]. However, the fundamental limitation for both ESTs and RNA-seq based transcriptome characterisation is the incomplete nature of their sequencing products, which require assembly. Full-length transcripts from RNA-seq are a very small proportion of the assembled transcripts, and mis-assembly is a problem that introduces inaccuracy in gene structure characterisation. Short read length from Illumina next-generation sequencing, makes it very difficult, if not impossible to associate a particular splicing event with another, particularly for transcripts containing repetitive elements. This problem is exacerbated in species without a reference genome sequence for the prediction of gene models. Our results confirmed that the assembled transcripts from 454 and Illumina sequencing platforms were much shorter than non-assembled transcripts from PacBio Iso-Seq, and many more Iso-Seq sequencing products contained full-length ORFs. Therefore, for species without a reference genome sequence, PacBio Iso-Seq is a more affordable and superior strategy for the direct generation of a comprehensive transcriptome with accurate AS isoforms compared with short read transcriptome sequencing to annotate a novel whole-genome assembly.

The standard Iso-Seq bioinformatics pipeline has been developed and applied to the characterisation of transcriptomes of different species. However, all previous studies were reference-guided and use straightforward methods that depend on the availability of genome assemblies. We created a pipeline using publicly available tools to analyse PacBio Iso-Seq data without the aid of a reference genome sequence. We used this pipeline on *A. membranaceus*, and obtained comprehensive transcriptomes from two different tissues. We were also able to detect different transcription isoforms without a reference genome. The effectiveness of this pipeline on *A. membranaceus* demonstrates that it can be used to characterise full-length transcriptomes in other species also lacking a reference genome assembly.

Compared with previous gene discovery studies in *A. membranaceus* using cDNA cloning or next-generation sequencing [19,20], we produced a more comprehensive transcriptome data set with the following features: First, many more full-length (with completed ORFs) transcripts were generated in our UniTransModels, providing a ‘plug-and-play’ resource that can be directly used for down-stream genetic functional studies without additional PCR amplification to get a complete transcript. Second, we were also able to detect splicing isoforms. The high proportion of transcripts with multiple splicing isoforms suggests a high degree of transcriptome complexity in *A. membranaceus*. We also found that there were different transcript isoforms in different tissues, which probably correspond to tissue-specific functions. Further studies are required to investigate the biological functions of these tissue-specific isoforms. For example, high-throughput next-generation sequencing from individual tissues could be used to validate the expression of different transcript isoforms. Third, lncRNAs were found in both tissues. Although only a minority of lncRNAs could be annotated with known RNA motifs, these still provide a useful resource for understanding the potential functions of lncRNAs in *A. membranaceus*. Finally, we re-characterised the key genes in the biosynthesis of several bioactive compounds from *A. membranaceus*. We also identified splicing isoforms for these genes, which will further our understanding of the tissue-specific regulation of these biosynthetic pathways.

In conclusion, we developed a robust computational pipeline to characterise the full-length transcriptome in two tissues of *A. membranaceus* using PacBio Iso-Seq. This study not only provides a practical guide for the analysis of full-length transcripts in species lacking a genome assembly, but also contributes a novel, valuable genetic resource for future research in gene discovery, molecular breeding and metabolic engineering of bioactive compounds in *A. membranaceus*.

## Materials and methods

### Sample collection and RNA isolation

Leaves and taproots of 17-month-old *A. membranaceus* were harvested and washed with distilled water at the Waite campus of The University of Adelaide, and then snap-frozen in liquid nitrogen. RNA extraction was performed using Spectrum Plant Total RNA Kit (Sigma-Aldrich, Sydney, NSW, Australia) following the manufacturer’s instructions.

### cDNA construction and PacBio Iso-Seq

The quality of extracted RNAs was evaluated using an Agilent 2100 Bioanalyzer (SA Pathology, Adelaide, SA, Australia). High-quality RNAs (RNA integrity number >7.0) were used for cDNA synthesis and library construction using SMART PCR cDNA kit (Clontech, Mountain View, CA, USA). The first strand cDNA was synthesised from total RNA by using SMARTScribe Reverse Transcriptase (Clontech), SMARTer II A Oligonucleotide (Clontech) and 3′ SMART CDS Primer II A (Clontech). The second strand was synthesised and amplified with 5′ PCR Primer II A. In total, 400 µl of cDNA for each sample was prepared for PacBio Iso-Seq library preparation and SMRT sequencing. PacBio Iso-Seq libraries were prepared and sequenced by DNA Link using the RS II platform with P6-C4 chemistry (DNA Link, Seoul, South Korea). Size selection was carried out on a BluePippin (Sage Science, Beverly, MA, USA) with two size bins for each sample: 1–2 and 2–3 kb. Three and five SMRT cells were used for sequencing 1–2 kb and 2–3 kb libraries for leaf or root samples, respectively.

### Iso-Seq data processing with standard bioinformatics pipeline

The standard RS_Iso-Seq protocol (SMRT Analysis 2.3) was used to process raw sequencing data. In summary, ROIs from raw data were called and separated into full-length and non-full-length based on primer and polyA tail detection with ‘pbtranscript.py classify’. Full-length ROIs were clustered and assembled into consensus sequences with isoform-level clustering. Subsequently, consensus sequences were polished with non-full-length ROIs and categorised as HQ (above 99% accuracy) or LQ full-length polished consensus transcripts using Quiver.

### Error correction of Iso-Seq consensus transcripts

Error correction for both HQ and LQ full-length polished consensus transcripts was performed with ‘proovread’ using an Illumina RNA-seq data set consisting of more than 13 million paired-end reads collected from shoot (no flower) and root tissues of *A. membranaceus* (Accession number: ERX651043, DDBJ) [27].

### Full-length unique transcript model reconstruction

Error-corrected HQ and LQ full-length polished consensus transcripts were merged and redundancy removed by using ‘cd-hit-est’ from the CD-HITv4.6 package with the following parameters: -c 0.99 –G 0 –aL 0.00 –aS 0.99 –AS 30 -M 0 –d 0 –p 1 [31]. The non-redundant transcripts were processed with Coding GENome reconstruction Tool (Cogent v1.4, https://github.com/Magdoll/Cogent). In general, Cogent firstly creates the k-mer profile of non-redundant transcripts and calculates pairwise distances and then clusters transcripts into families based on their k-mer similarity. Each transcript family is further reconstructed into one or several unique transcript model(s) (referred to as UniTransModels) using a De Bruijn graph method.

### Isoform identification

Error-corrected non-redundant transcripts (transcripts before Cogent reconstruction) were mapped to UniTransModels using GMAP v2014-08-04 [32]. Splicing junctions for transcripts mapped to the same UniTransModels were examined, and transcripts with the same splicing junctions were collapsed. Collapsed transcripts with different splicing junctions were identified as transcription isoforms of UniTransModels. AS events were detected with SUPPA using default settings [33].

### Next-generation sequencing data analysis

Raw reads from the Illumina RNA-seq data set were trimmed using trim_galore v0.3.7 (parameters: –stringency 6 –paired) to remove adaptor and LQ sequences. Cleaned reads were used for error correction as mentioned above and *de novo* assembly using trinityrnaseq-2.0.6 with default parameters [34]. *De novo* assembled long transcripts were annotated with Trinotate [34]. Full-length analysis was performed with ‘analyze_blastPlus_topHit_coverage.pl’ from the trinityrnaseq-2.0.6 package [34].

### Functional annotation and GO over-representation analyses

UniTransModels were used to search four well-characterised protein databases: UniProtKB_Viridiplantae (including all green plant curated protein entries), UniProtKB_MEDTR (including all medicago curated protein entries), UniProtKB_SOYBN (including all soybean curated protein entries) and curated soybean reference protein annotation (Wm82.a2.v1) using BLASTX (NCBI-BLAST v2.2.27+) with the following parameters: -max_target_seqs 1 -outfmt 6 -evalue 1e-3 [35,36]. The annotation based on the best hit from UniProtKB_SOYBN was then used to classify UniTransModels into GO functional terms with PANTHER [37]. KEGG classification of UniTransModels was performed with annotation based on the best hit from UniProtKB_SOYBN using the R package ‘KEGGREST’ [38].

GO over-representation analysis of tissue-specific UniTransModels was performed using agriGO webtools based on the best hit from soybean reference protein annotation (Wm82.a2.v1) with the following thresholds: false discovery rate<0.05, minimum genes as 5 [39].

### LncRNA identification

First, UniTransModels with hits in any of the four above-mentioned protein databases were removed. ORF of unannotated UniTransModels were subsequently identified and UniTransModels with internal ORFs longer than 100 amino acids (aa) or 50 aa at end(s) were filtered out. ORF-filtered UniTransModels were evaluated with Coding Potential Calculator v0.9r2 and transcripts that passed the evaluation were annotated as lncRNAs [28].

### Accession numbers

The sample information was deposited as BioSample metadata in the NCBI BioSample database (http://www.ncbi.nlm.nih.gov/biosample/) under accession numbers: SAMN06603371, SAMN06603372 and raw consensus transcripts from PacBio Iso-Seq were deposited and are available in the NCBI Sequence Read Archive database under accession numbers: SRR5343992, SRR5343993. The sequences of UniTransModels and isoforms, as well as lncRNAs were deposited and are available in the Dryad Digital Repository (http://datadryad.org) under doi: http://dx.doi.org/10.5061/dryad.4sf85.

## Figures and Tables

**Table 1:** Summary of consensus transcripts after standard Iso-Seq bioinformatics pipeline

*Tissue*	*Number of high-quality consensus transcripts*	*Number of low-quality consensus transcripts*	*Total number of consensus transcripts*	*Average consensus transcript length (nt)*
Leaf	75 816	39 909	115 725	2 336
Root	73 755	28 579	102 334	2 218
