# Supplementary material for: Long read reference genome-free reconstruction of a full-length transcriptome from Astragalus membranaceus reveals transcript variants involved in bioactive compound biosynthesis
Source: Cell Discov. 2017 Aug 29;3:17031–. doi: 10.1038/celldisc.2017.31 (PMC5573880; doi:10.1038/celldisc.2017.31)
Supplement: Supplementary Figures [file celldisc201731-s1.pdf]

Supplementary figures:

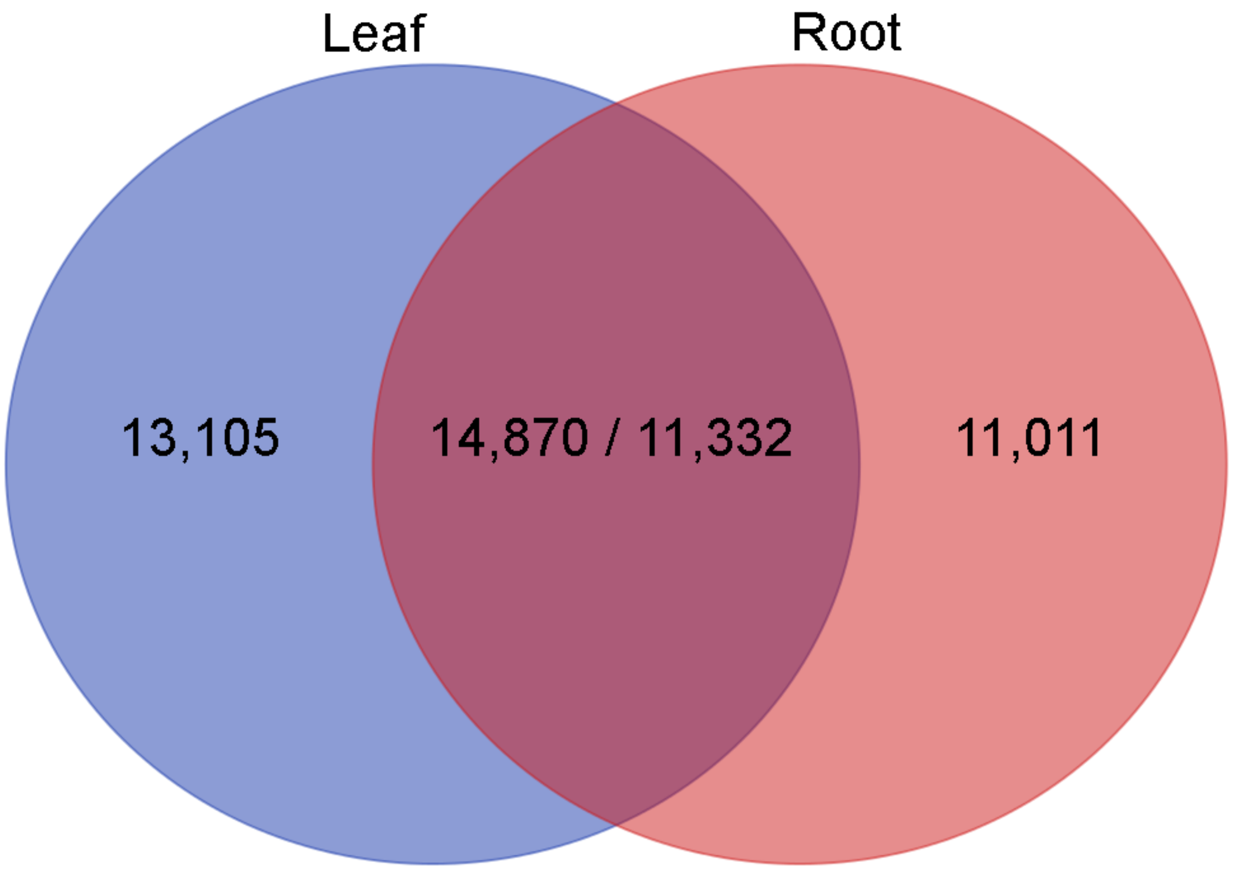

Supplementary Figure S1, Number of common UniTransModels in leaf and root tissues. There were 14,870 UniTransModels in leaf that shared high sequence similarity with 11,332 UniTransModes in root, and were therefore considered as common UniTransModels. There were 13,105 leaf specific UniTransModels and 11,011 root specific UniTransModels.

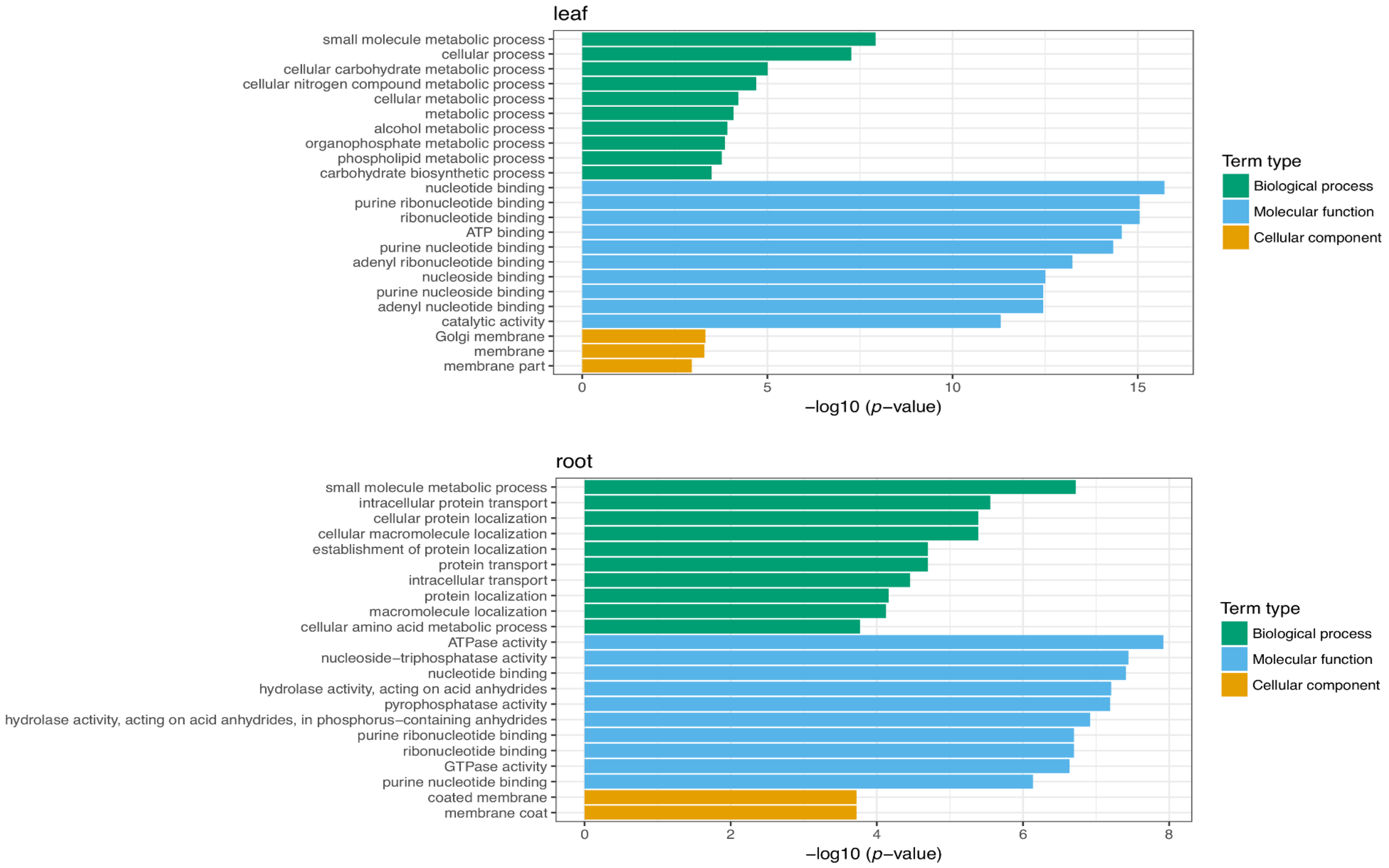

Supplementary Figure S2, GO over-representation analysis of tissue-specific genes in leaf and root. Significantly over-represented GO terms were selected using Hypergeometric test with FDR < 0.05. Only the 10 most significant over-represented GO terms were visualised for "Biological process" and "Molecular function" in the barplot.

(A)

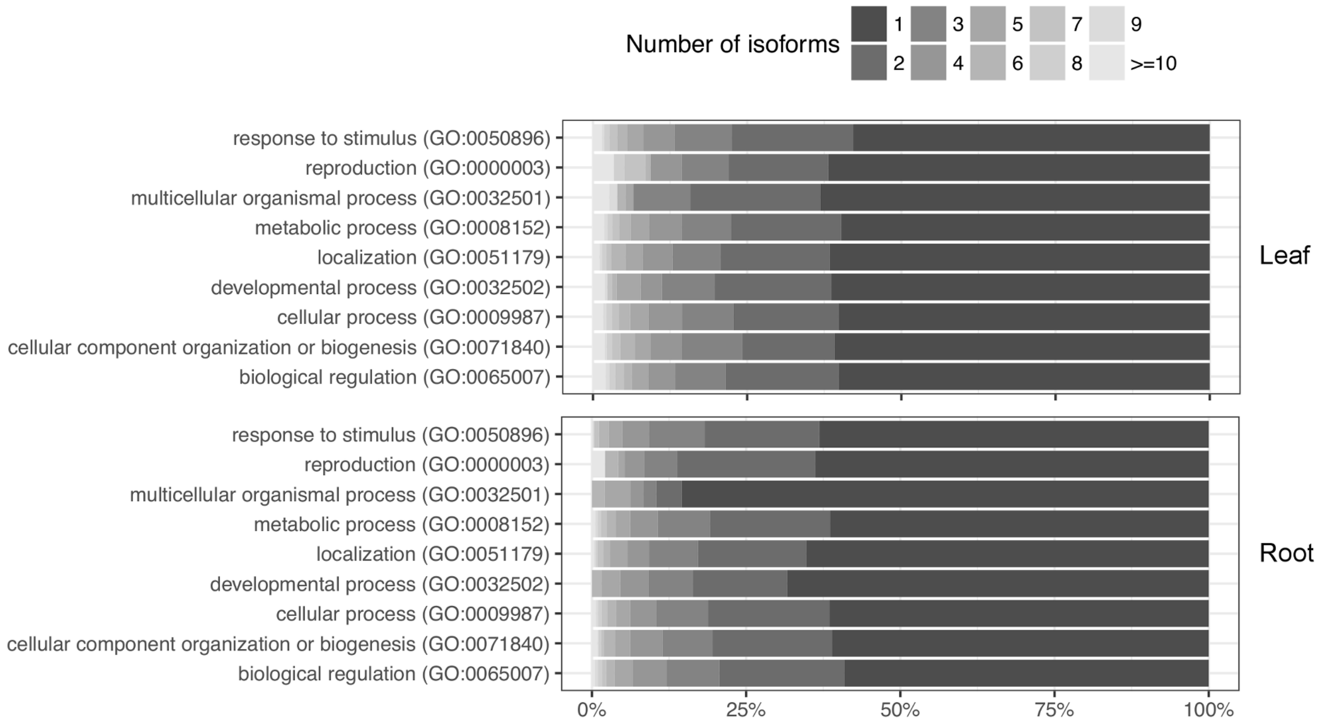

(B)

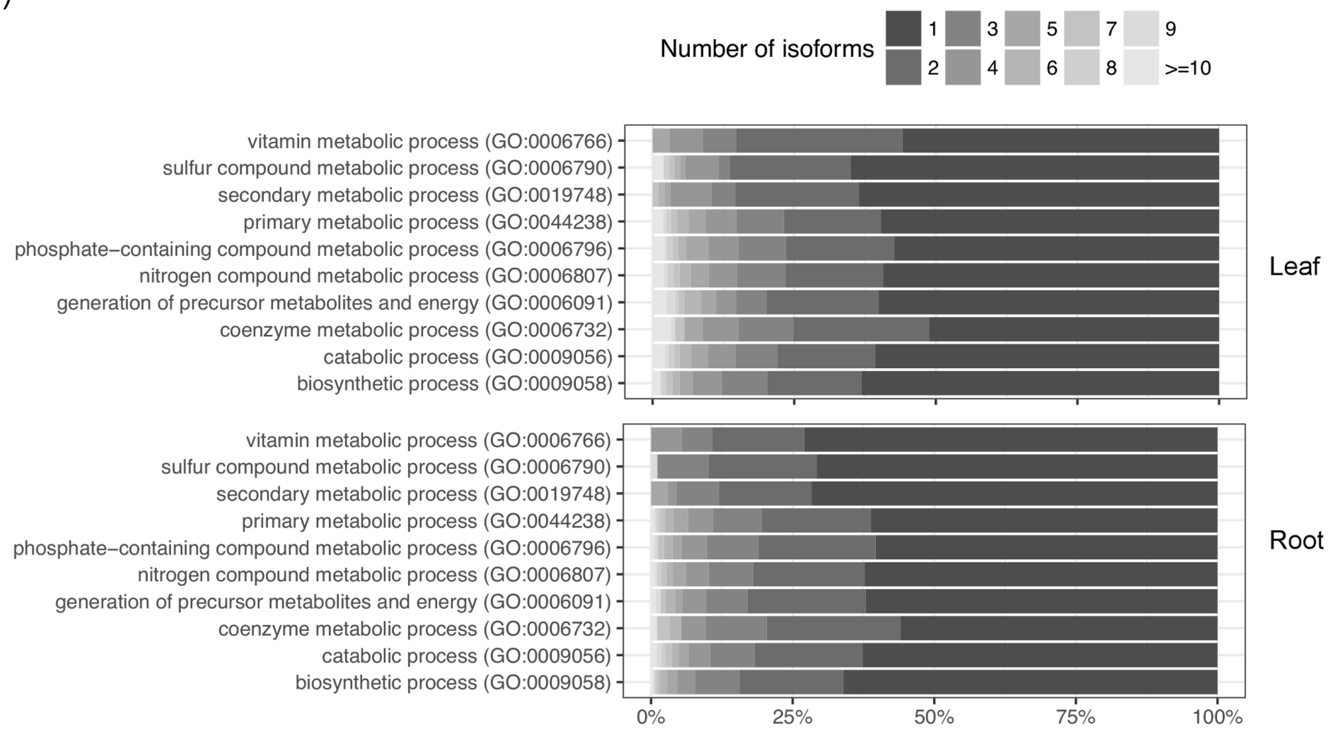

Supplementary Figure S3, Distribution of numbers of isoforms for genes in different GO biological process functional terms. (A) Distribution of numbers of isoforms for genes in top-level GO biological process terms. Only terms with more than 10 genes were considered. **(B)** Distribution of number of isoforms for genes in sub-level GO functional terms under the top-level of "metabolic process". Only terms with more than 10 genes were considered.

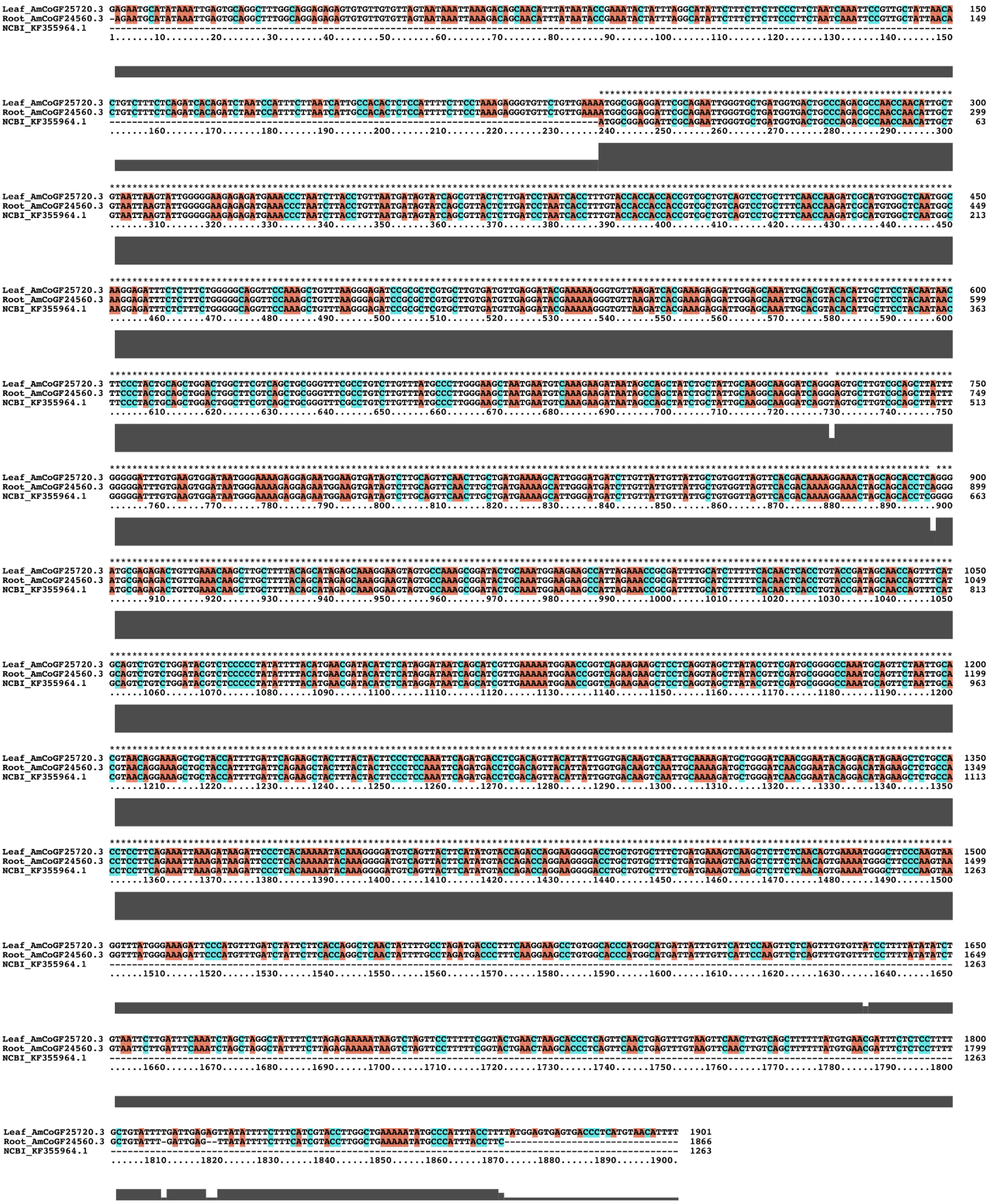

Supplementary Figure S4, Sequence alignment of "Isoform 3" in leaf and "Isoform 3" in root of *AmMVD gene* compared to NCBI reference (Accession number: KF355964).

Table S1, Summary of ROIs of individual SMRT cells for leaf and root.

Table S2, Annotation of UniTransModels in two tissues.

Table S3, Summary of numbers of UniTransModels annotated with different protein databases.

Tables S4, Known RNA motifs annotated in *A. membranaceus* lncRNAs.

Tables S5, Summary of re-characterisation and transcription isoforms of genes involved in AST biosynthesis.

Table S6, Summary of re-characterisation and transcription isoforms of genes involved in Calycosin and CG biosynthesis.
